# Triblock Polyampholyte‐Based Nanovesicles for Targeted Spleen Delivery

**DOI:** 10.1002/mabi.202500147

**Published:** 2025-05-22

**Authors:** Takayoshi Watanabe, Keita Masuda, Pengwen Chen, Horacio Cabral

**Affiliations:** ^1^ Department of Bioengineering Graduate School of Engineering The University of Tokyo 7‐3‐1 Hongo, Bunkyo‐ku Tokyo 113‐0033 Japan

**Keywords:** biodistribution, nanocarrier, polyion complex, polymeric vesicles, triblock copolymer

## Abstract

Polymeric vesicles are a promising platform for targeted drug delivery. In this study, nanovesicles are developed using triblock polyampholytes composed of neutral poly(ethylene glycol), cationic poly(L‐lysine), and anionic poly(α,β‐aspartic acid) segments (PEG‐PLys‐PAsp) poly(aspartate) segments. By controlling the polymerization degree of these cationic and anionic segments, narrowly distributed nanovesicles are successfully assembled with a hydrodynamic diameter of ≈140 nm. The membrane thickness of the nanovesicles is around 15 nm, corresponding to a uniform polyion complex layer. Cross‐linking the membrane of the nanovesicles via amide bonds enhance their stability in physiological salt and temperature conditions. In vivo, the cross‐linked nanovesicles exhibit prolonged blood circulation and selective accumulation in the spleen after intravenous injection in mice. This approach demonstrates the potential of polyampholyte‐based nanovesicles (TPBV) for targeted drug delivery applications to the spleen.

## Graphical abstract

Polymeric nanovesicles, assembled from triblock polyampholytes, exhibit unilamellar structure and enhanced stability through membrane cross‐linking. These nanovesicles demonstrate prolonged blood circulation and selective accumulation in the spleen after intravenous injection in mice, highlighting their potential for targeted drug delivery applications.

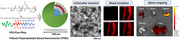

## 1 Introduction

Precisely engineered supramolecular structures have attracted high attention in both basic research and the development of functional biomaterials.^[^ ^1^, ^2^ ^]^ Among these structures, nano‐ and micro‐vesicles offer significant advantages for compartmentalizing fragile functional substances.^[^ ^3^, ^4^, ^5^, ^6^, ^7^ ^]^ The ability of these vesicles to encapsulate fragile materials has helped preserve or even enhance their functionality.^[^ ^8^, ^9^, ^10^, ^11^ ^]^ Particularly, synthetic vesicles formed from amphiphilic block copolymers, i.e., polymersomes,^[^ ^12^ ^]^ have shown high protection of the encapsulated molecules and robust mechanical properties due to their thick membrane structures, making them a promising tool for delivery applications.^[^ ^13^, ^14^, ^15^, ^16^ ^]^ However, challenges remain, particularly regarding biocompatibility and encapsulation, which present significant barriers to their widespread use. Moreover, the process of creating these vesicles typically requires precise adjustments to polymer structures, template preformation, and the use of organic solvents to control the vesicle size at the nanometer scale.^[^ ^17^ ^]^ The complexity of these processes makes the scalable production of polymersomes a challenge.

Polyion complex (PIC)‐based vesicles, known as PICsomes, offer several advantages compared to traditional polymersomes. PICsomes can be self‐assembled in aqueous buffers through electrostatic interactions between oppositely charged polymers, eliminating the need for organic solvents.^[^ ^18^, ^19^ ^]^ Notably, PICsomes enable efficient encapsulation of water‐soluble macromolecules in their aqueous inner compartment, while allowing controlled permeability of the vesicular membrane.^[^ ^20^, ^21^ ^]^ Despite these advantages, the current formulations of PICsomes require precise mixing of oppositely charged block catiomers and aniomers for achieving uniform and stable structures.

**Scheme 1:**
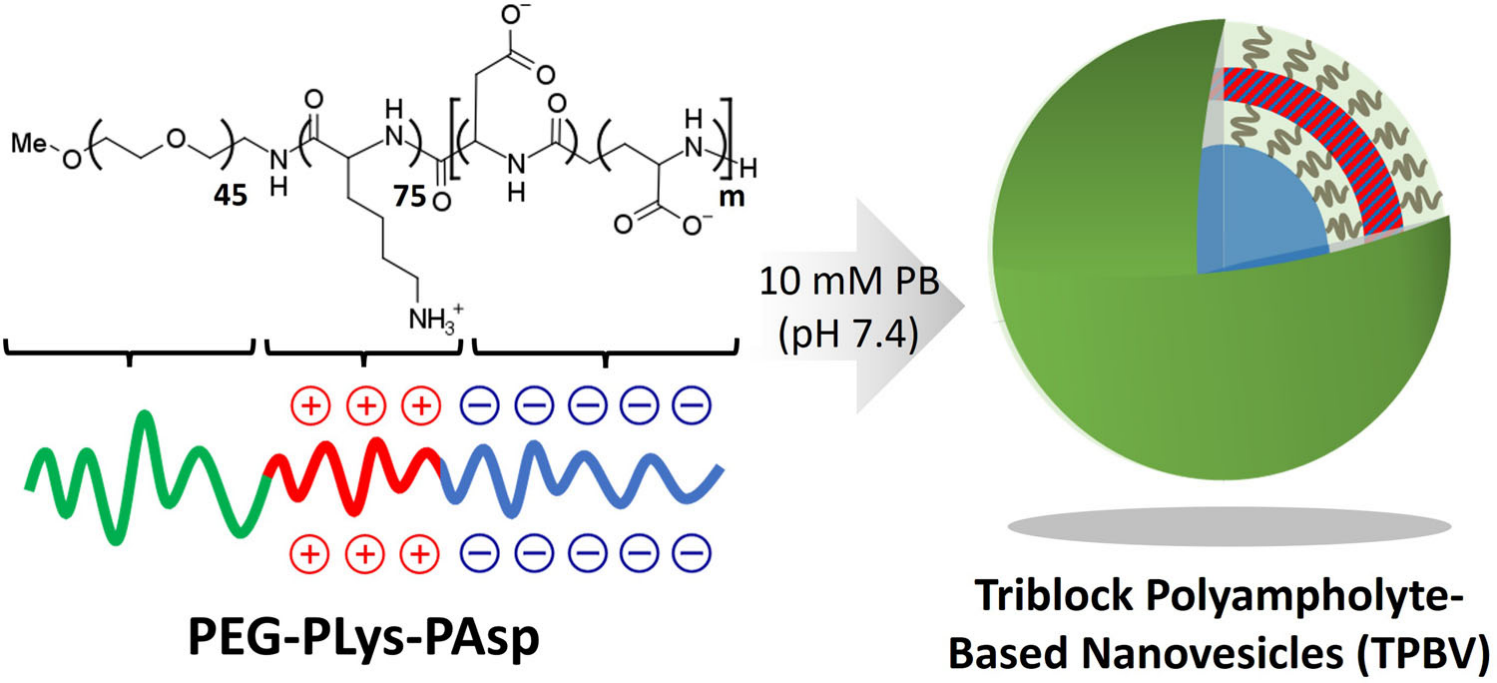
Preparation of nanovesicles from poly(ethylene glycol)‐poly(L‐lysine)‐poly(α,β‐aspartic acid) (PEG‐PLys‐PAsp). By engineering the length of the charged segments, the triblock polyampholyte spontaneously assembled into nanovesicles in aqueous conditions after dissolution.

Herein, we focused on developing nanovesicles based on polyampholytes that can spontaneously self‐assemble when dissolved in aqueous media. As polyampholytes inherently contain both positive and negative charges within their structure, they eliminate the need for mixing separate components, like traditional PICsomes.^[^ ^22^, ^23^ ^]^ To achieve this, we used polyampholytes composed of neutral PEG block, cationic poly(L‐lysine), and anionic poly(aspartate) segments (**Scheme** **1**). By controlling the polymerization degree of the cationic and anionic segments, we successfully constructed triblock polyampholyte‐based nanovesicles (TPBV) with promising features for in vivo application, such as prolonged circulation in the bloodstream and targeted delivery to the spleen.

## 2 Experimental Section

### 2.1 Materials

α‐methoxy‐ω‐amino‐poly(ethylene glycol) (MeO‐PEG‐NH_2_; M_w_ = 2,000 g mol^−1^) was purchased from Nippon Oil and Fats Co., Ltd. (Tokyo, Japan). *β*‐benzyl‐L‐aspartate *N*‐carboxy‐anhydride (BLA‐NCA) was purchased from Santa Cruz Biotechnology (Texas, United States). *N*‐trifluoroacetyl‐L‐lysine *N*‐carboxyanhydride (Lys(TFA)‐NCA) was bought from Chuo Kasei Co., Inc (Tokyo, Japan). Deuterium oxide (99.8 atom%D) was purchased from Tokyo Chemical Industry Co. Ltd. (Tokyo, Japan). Omnipore 10 µm PTFE Membrane was purchased by Merck Millipore Ltd. (Tullagreen, Ireland). DMSO‐*d_6_* was purchased from Sigma–Aldrich (St. Louis, MO, USA). Super Dehydrated N,N‐Dimethylformamide and 1‐Ethyl‐3‐(3‐dimethylaminopropyl) carbodiimide hydrochloride (EDC‐HCl) were purchased from Wako Pure Chemical Industries (Osaka, Japan). Sodium hydroxide solution (1 mol L^−1^), 1,4‐Dioxane, Lithium Chloride, and D‐PBS(‐) were purchased from Fujifilm Wako. Cy5 mono‐reactive dye pack was purchased from GE Healthcare (Chicago, USA). N,N‐Dimethylformamide for HPLC was purchased from Junsei Chemical Co. Ltd. (Tokyo, Japan). PEG nominal 1.5 k, 7 k, 10 k, 13 k, and 20 kDa were purchased from Agilent Technologies (Cheadle, UK). TSKstandard Polyethylene oxide 24 k and 50 kDa were purchased from TOSOH (Tokyo, Japan). Dialysis membranes were purchased from Spectrum Laboratories Inc. (Rancho Dominguez, CA, USA). Vivaspin 6 centrifugal filter units with a molecular weight cutoff (MWCO) of 300000 were purchased from Sartorius (Gottingen, Germany).

### 2.2 Animals

BALB/c nude mice (female; 3 weeks old) were purchased from Charles River Japan (Kanagawa, Japan). All animal experiments were approved by the Ethical Committee of the University of Tokyo (Number: AF2023E003).

### 2.3 Instruments

Proton nuclear magnetic resonance (^1^H‐NMR) spectra were obtained using a JEOL ECS‐400 spectrometer (JOEL Ltd., Japan) with a frequency of 400 MHz, and chemical shift was calculated as parts per million (ppm). Molecular weight distribution of the polymers was determined by gel permeation chromatography (GPC) that was carried out on a JASCO LC‐EXTREMA system (JASCO, Japan) equipped with a size exclusion column TSKgel GMH_HR_‐M(S) (TOSOH, Japan) and Superdex 200–10/300GL (GE Healthcare, USA). The Z‐average diameter and zeta‐potential were measured through dynamic light scattering (DLS) and laser Doppler electrophoresis, respectively, at 25 °C using a Zetasizer Nano‐ZS instrument (Malvern Instruments, Malvern, UK) equipped with a He–Ne ion laser (λ 517 nm). A scattering angle of 173° was used in all measurements. Intravital microcopic observations were performed using an A1R confocal laser scanning microscope (Nikon Co., Tokyo, Japan) connected to an upright Eclipse FN1 (Nikon Co.).

### 2.4 Synthesis of Triblock Copolymer

**Scheme 2:**
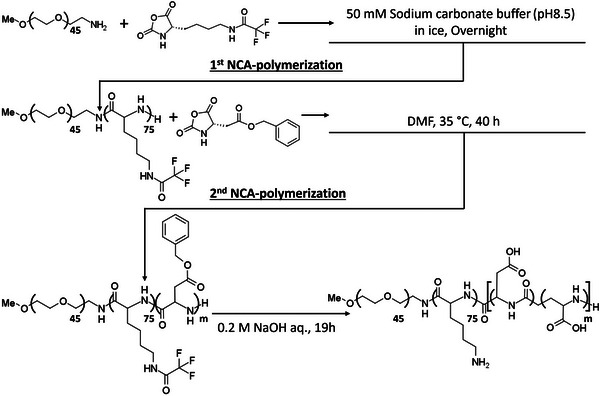
Preparation of the triblock polyampholyte poly(ethylene glycol)‐poly(L‐lysine)‐poly(α,β‐aspartic acid).

The synthesis of a series of poly(ethylene glycol)‐poly(N‐trifluoroacetyl‐L‐lysine)‐poly(β‐benzyl‐L‐aspartate) (PEG‐PLys(TFA)‐PBLA) triblock copolymers was done by *N*‐carboxyanhydride (NCA) ring‐opening polymerization (ROP), as previously reported,^[^ ^24^, ^25^ ^]^ but with modifications (**Scheme** **2**). The initiator used in the first ROP step was MeO‐PEG‐NH_2_ to produce PEG‐poly(L‐lysine) diblock copolymer (PEG‐PLys). At first, MeO‐PEG‐NH_2_ at 87.85 mg was dissolved in 10 mL of 50 mm sodium carbonate buffer (pH 8.5) on ice. *N*‐trifluoroacetyl‐L‐lysine *N‐*carboxyanhydride (Lys‐(TFA)‐NCA) (873.7 mg; 75 molar equivalent to MeO‐PEG‐NH_2_) was added to the MeO‐PEG‐NH_2_ solution, and the mixture was then added to the MeO‐PEG‐NH_2_ solution while stirring rapidly in an ice bath. The mixture was left to react for 12 h and dialyzed against deionized water using a membrane with a MWCO of 6000–8,000 Da for 2 days to remove the sodium carbonate buffer. The solution was then lyophilized. The freeze‐dried diblock polymer was dissolved in 12 mL of DMF and dropped into 200 mL of diethyl ether, followed by filtration of the precipitated polymer, and drying under vacuum to remove unreacted NCA‐Lys(TFA). The degree of polymerization (DP) of the Lys(TFA) block was analyzed from ^1^H‐NMR by comparing the characteristic peaks of ─O─CH_2_─CH_2_─ in the PEG block with the peaks of ─CH_2_─CH_2_─CH_2_─ in the Lys(TFA) side chain. The analysis was conducted in DMSO*‐d*_6_ at 80 °C. The GPC of the diblock polymer was done in 10 mm lithium chloride with N,N‐Dimethylformamide using the GPC system mentioned above at a flow rate of 0.75 mL min^−1^ at 40 °C.

The obtained poly(ethylene glycol)‐poly(*N*‐trifluoroacetyl‐L‐lysine) (PEG‐PLys(TFA)‐NH_2_) was subsequently used as an initiator for the second ROP of BLA‐NCA to obtain PEG‐PLys(TFA)‐PBLA. The reaction was done as follows: after dissolving PEG‐PLys(TFA)‐NH_2_ (527.56 mg) in 30 mL of methanol, it was subsequently evaporated. Then, the polymer was freeze‐dried with benzene and 1,4‐Dioxane. The lyophilized polymer was subsequently dissolved in 10 mL of super dehydrated DMF. BLA‐NCA (0.263, 0.396, 0.659, 0.920, and 1.186 g, i.e., 37.85, 56.99, 94.84, 132.4 and 170.68 molar equivalent to PEG‐PLys(TFA)‐NH_2_) was dissolved in 20 mL of super dehydrated DMF and introduced to the PEG‐PLys(TFA)‐NH_2_ solution under an Ar atmosphere. The mixture was kept at 35 °C for 40 h. The mixture was then precipitated in 300 mL of diethyl ether to remove unreacted NCA‐BLA and finally dried in vacuo. The composition of the BLA in the polymer was analyzed by ^1^H NMR spectroscopy in DMSO‐*d*_6_ at 80 °C.

PEG‐PLys(TFA)‐PBLA was deprotected in 20 mL of 0.2 m NaOH solution at 25 °C for 19 h. The polymer was dialyzed against 1 mol L^−1^ NaCl solution and deionized water. The solution was then freeze‐dried to obtain poly(ethylene glycol)‐poly(L‐lysine)‐poly(α,β‐aspartic acid) (PEG‐PLys‐PAsp). The final DP of the Lys block was analyzed from ^1^H‐NMR by comparing the characteristic peaks of ─O─CH_2_─CH_2_─ in the PEG block with the peaks of ─CH_2_─CH_2_─CH_2_─ in the Lys side chain after the deprotection. In addition, the DP of the Asp block was analyzed from ^1^H‐NMR, likewise by comparing the characteristic peaks of ─O─CH_2_─CH_2_─ in the PEG block with the peaks of ─CH─ in the side chain of Asp. The characterization was conducted in D_2_O with 2.0 m NaCl at 80 °C. The GPC of the triblock copolymers was done in 10 mm phosphate buffer with 2 m NaCl (pH 7.4) using the HPLC system mentioned above at a flow rate of 0.75 mL min^−1^ at room temperature.

### 2.5 Nanovesicle Preparation and Characterization

The series of triblock copolymers was dissolved in 10 mm phosphate buffer (PB) (pH 7.4) at 10 mg mL^−1^ on ice and followed by vortexing to form polyion complexes between the P(Lys) and P(Asp) segments. The size of the polyion complexes and zeta‐potential were evaluated by Zetasizer Nano ZS. In addition, the nano‐structures were observed using transmission electron microscopy (TEM). The experiment was performed with a JEOL Bio‐TEM JEM‐1400 (JEOL, Tokyo, Japan) microscope operating at 75 kV. Copper grids were coated with a thin film of collodion, followed by a subsequent coating with carbon. The sample solution (2 µL) was placed on the grids. Then, it was briefly stained by depositing a drop of a 50% ethanol solution containing 2 wt.% uranyl acetate onto the surface of the sample‐loaded grid, which was then dried at room temperature.

For preparing cross‐linked polyion complexes, 1, 5 and 10 mg mL^−1^ of PEG‐PLys_75_‐PAsp_130_ solution in PB buffer (357 µL) was added to a 10 mg mL^−1^ of EDC HCl solution (357 µL) at 4 °C. After 12 h, the solution was purified by dialysis using a polyethersulfone ultrafiltration membrane (MWCO: 30 0000). The size of the cross‐linked polyion complexes was evaluated by DLS at each condition and the nano‐structure was observed by normal TEM. It was performed on a JEOL Bio‐TEM JEM‐1400 (JEOL, Tokyo, Japan) microscope operating at 75 kV. Copper grids were coated with a thin film of collodion, followed by a subsequent coating with carbon. The sample solution (2 µL) was placed on the grids. Samples were stained by deposition of a drop of a 50% ethanol solution containing 2 wt.% uranyl acetate onto the surface of the sample‐loaded grid and dried at room temperature.

The cross‐linked nanovesicle was also measured by cryogenic phase contrast transmission electron microscopy (cryo‐TEM). Briefly, the grid was treated by glow discharge. Then, a 2 µL drop of the nanovesicle solution was placed on the micro‐grid. The drop was blotted with filter paper until it was reduced to a thin film. The sample was then vitrified by rapid immersion into liquid ethane near its freezing point. Vitrification was performed on an EM CPC cryo‐station (Leica Microsystems, Vienna, Austria). The vitrified specimen was observed by a JEM 2100‐F transmission electron microscope (JEOL, Tokyo, Japan).

### 2.6 In Vitro Characterization of Cross‐Linked Nanovesicles

In vitro stability of the cross‐linked nanovesicles was evaluated in the presence of FBS. The nanovesicles were incubated in D‐PBS containing 10% FBS at 37 °C for 24 h. During incubation, the samples were consecutively analyzed by DLS. The size and PDI of the nanovesicles were normalized to the size and the PDI before incubation to assess the stability.

### 2.7 Evaluation of Blood Circulation and Biodistribution

To evaluate the blood circulation and biodistribution, the nanovesicle was labeled with Cy5‐NHS, as follows: the cross‐linked nanovesicle solution (10 mg ml^−1^; 500 µL) was mixed with Cy5‐NHS in DMSO (500 µL) and reacted at 4 °C for 24 h. The reaction mixture was dialyzed against deionized water (MWCO: 6000‐8000) for 7 days. The solution was purified by filtration through a 0.22 µm membrane filter to remove dust and concentrated to 1 mg mL^−1^ by ultrafiltration membrane in D‐PBS. The Cy5‐labeling was analyzed by aqueous phase GPC (10 mm phosphate buffer with 500 mm NaCl at pH 7.4; eluent, flow rate 0.75 mL min^−1^; room temperature). The Cy5‐labeled nanovesicle (100 µL; 100 µg µL^−1^ of PEG‐Plys_75_‐PAsp_130_) was injected through the tail vein of mice. The fluorescence intensity in the earlobe vein and skin was monitored continuously by Nikon A1R intravital confocal laser scanning microscopy (IV‐CLSM). Twelve hours after injection, the mice were euthanized, and the kidney, liver, lung, heart, and spleen were collected and imaged ex vivo by IVIS (filters: E_x_640/E_m_680).

## 3 Results and Discussion

### 3.1 Synthesis of Triblock Copolymers

PEG‐PLys(TFA) block copolymer was successfully synthesized through ROP of Lys(TFA)‐NCA, initiated by the terminal primary amino group of MeO‐PEG‐NH_2_. After purifying the polymer by diethyl ether precipitation, the DP of lysine was determined by analyzing the ^1^H NMR proton ratios of ─OCH_2_─CH_2_─ (δ = 3.5 ppm) of PEG and ─CH_2_─CH_2_─CH_2_─ (δ = 1.2–1.8 ppm) of P(Lys). The DP of lysine was calculated as 75 (Figure S1, Supporting Information). In addition, the GPC result showed the successful synthesis of mono‐dispersed PEG‐P(Lys(TFA)) with a PDI of 1.02 (Figure S2, Supporting Information). To explore the impact of charge balance and interaction strength on nanostructure formation and stability, we synthesized a series of triblock copolymers with PLys(TFA) blocks having 75 units but varying lengths of the PBLA segment. In particular, previous reports demonstrated that homopolycations with a DP of 75 could form well‐defined lamellar structures with PEG‐polyanions, enabling the formation of stable PICsome.^[^ ^26^ ^]^ The obtained PEG‐PLys(TFA)‐NH_2_ was subsequently used as an initiator for the second ROP of BLA‐NCA, and the reaction was successfully synthesized in anhydrous DMF (Figure S3, Supporting Information). After removing the protecting TFA and BLA groups through alkaline hydrolysis, the DP was confirmed by ^1^H NMR using the proton ratios of ─OCH_2_─CH_2_─ (δ = 3.5 ppm) of PEG, ─CH_2_─CH_2_─CH_2_─ (δ = 1.2–1.8 ppm) of P(Lys) and ─CH─ (δ = 2.50–2.85 ppm) of P(Asp) in the side chain (Figure S4, Supporting Information). As a result, the DP of the P(Lys) block was determined to be 75 units, while the DPs of the P(Asp) segments were 37, 56, 92, 130, and 170 units (**Table** **1**). Thus, these polyampholytes contain different carboxylate to amine molar ratios ([carboxylate]/[amine] ([mol]/[mol], C/A) (Table 1). To analyze the molecular weight distribution, we performed GPC analysis in high salt buffer for disrupting the electrostatic interactions between the polyampholytes. The results showed that the polymers present unimodal peaks with narrow distribution (Figure S5, Supporting Information). The polydispersity indexes (M_w_/M_n_) are reported in Table 1.

**Table 1:** Characteristics of the series of poly(ethylene glycol)‐poly(L‐lysine)‐poly(α,β‐aspartic acid) (PEG‐PLys‐PAsp).

Name of polymer	Polymer characteristics				
	DP of PLys^a)^	DP of PAsp^a)^	C/A	M_w_/M_n_ ^b)^	M_n_ ^a)^
PEG‐PLys(TFA)_75_	75	–	–	–	18800
PEG‐PLys_75_‐PAsp_37_	75	37	0.5	1.02	13643
PEG‐PLys_75_‐PAsp_56_	75	56	0.8	1.02	15809
PEG‐PLys_75_‐PAsp_92_	75	92	1.2	1.05	19913
PEG‐PLys_75_‐PAsp_130_	75	130	1.7	1.02	24245
PEG‐PLys_75_‐PAsp_170_	75	170	2.3	1.04	28805

^a)^ determined by ^1^H‐NMR;

^b)^ determined by GPC.

### 3.2 Preparation and Characterization of Nanovesicles

**Figure 1:**
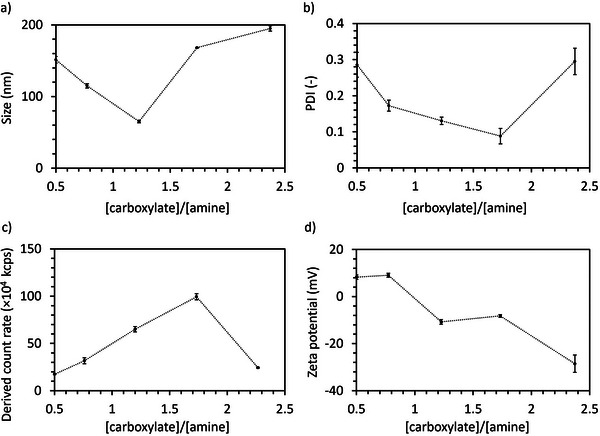
Characterization of complexes from the polyampholytes in 10 mm PB buffer (pH 7.4). a) Size, b) Polydispersity index (PDI), c) Derived count rate, and d) Zeta potential. Data shown as the mean ± S.D. (n = 3).

To study the assembly of the polyampholytes, we dissolved the polymers at 10 mg mL^−1^ in 10 mm phosphate buffer (pH 7.4). DLS results showed that the polyampholytes formed complexes larger than 30 nm at any C/A (**Figure** **1**a). Moreover, the complexes formed at C/A = 0.8, 1.2, and 1.7 also showed relatively low polydispersity index (PDI) (Figure 1b). The derived count rate of the complexes (Figure 1c), which correlates with a strong light scattering intensity from the particles, increased as the C/A increased. However, it abruptly declined at C/A = 2.3, indicating that this polyampholyte is unable to efficiently form stable complexes, probably due to the large negatively charged PAsp block. Notably, for a C/A ratio of 1.7, i.e., PEG‐PLys_75_‐PAsp_130_, the complexes showed the highest derived count rate and the lowest PDI. Moreover, the zeta‐potential of the complexes was measured by ELS. The results indicate that the zeta‐potential decreased from 9.5 mV for C/A = 0.5 to ‐33.3 mV for C/A = 2.3 (Figure 1d). Notably, increasing the amount of negatively charged groups in the complexes from C/A = 1.2 to C/A = 1.7 did not decrease the zeta‐potential, suggesting that the additional negative charges are effectively shielded by the PEG chains in the C/A = 1.7 formulation.

**Figure 2:**
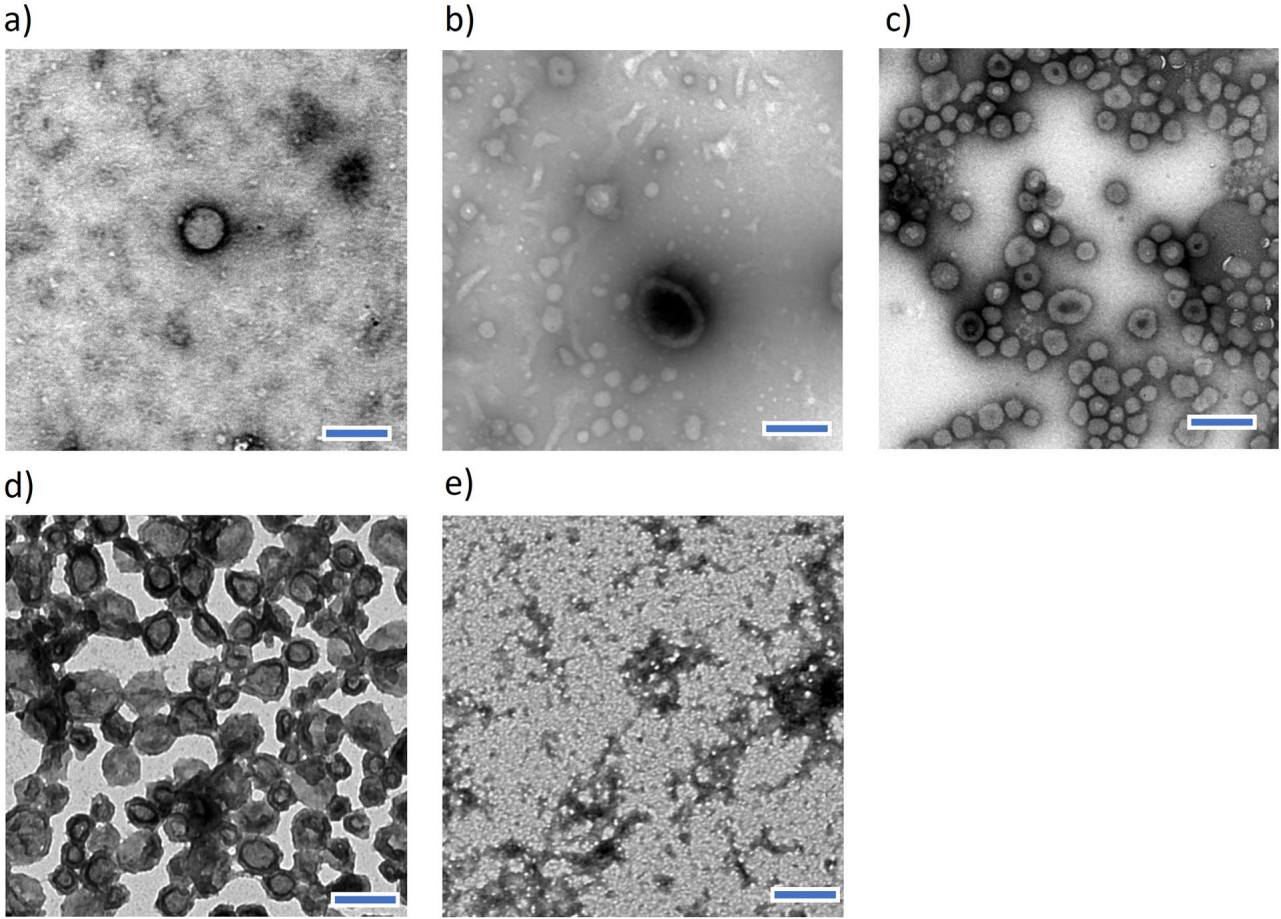
TEM observation of the complexes from the triblock polyampholytes (pH 7.4) at C/A a) 0.5, b) 0.8, c) 1.2, d) 1.7, and e) 2.3. All scale bars are 200 nm.

To understand the structure of the complexes formed by the different polyampholytes, we took TEM images of each complex (**Figure** **2**a–e). The formation of nanovesicles was evident only for C/A = 1.7 (Figure 2c), showing clear ring‐like structures. On the other hand, the other C/A were not able to consistently assemble into vesicular structures, as visualized by a mixture of small complexes and nanovesicles. These results indicate that shorter PAsp blocks would lead to weaker complexation and less stable vesicles, while longer PAsp chains could promote stronger interactions and more robust vesicle structures due to improved charge compensation.^[^ ^27^ ^]^ However, extending the PAsp block too much would lead to an imbalance in the charge density, causing excessive electrostatic repulsion and preventing the association of the vesicular structure.^[^ ^28^ ^]^ Therefore, we selected C/A = 1.7 as optimal conditions for assembling nanovesicles and tested the temperature stability. The resulting nanovesicle at C/A = 1.7 was stable at 4 °C, but gradually increased its size when it was incubated at room temperature (Figure S6, Supporting Information). This observation suggests coarsening at room temperature facilitated high dynamic behavior for the nanovesicles, such as diffusion‐limited coarsening (or Ostwald ripening) or Brownian coalescence (or fusion).^[^ ^28^, ^29^ ^]^ At low temperatures, these dynamic properties are reduced, allowing the nanovesicle shape to be maintained. These findings indicate that the nanovesicle should be stabilized for further characterization.

### 3.3 Preparation and Characterization of Cross‐Linked Nanovesicles

**Figure 3:**
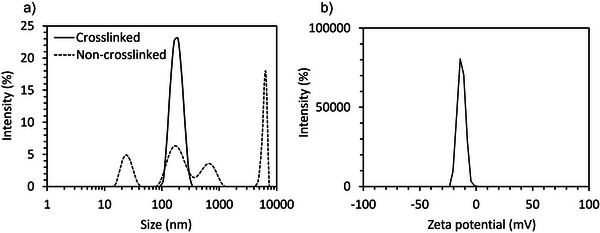
Characterization of nanovesicles after cross‐linking with EDC. a) DLS histogram in 10 mm PB buffer (pH 7.4, 2 m NaCl) of nanovesicles with and without cross‐linking. b) Zeta potential in 10 mm PB buffer (pH 7.4, 0 m NaCl).

**Figure 4:**
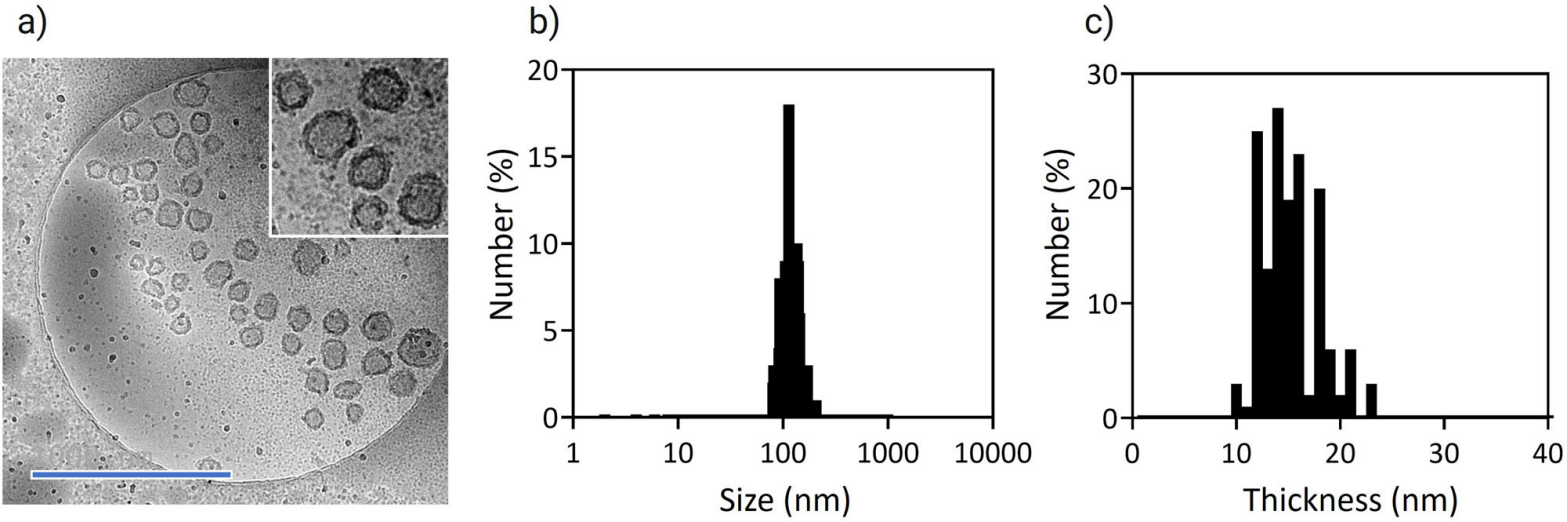
Cryo‐TEM observation of nanovesicles. a) Representative cryo‐TEM image of vesicle after cross‐linking treatment. The scale bar is 1000 nm. b) Size and c) thickness of nanovesicles calculated from cryo‐TEM images (n = 150 nanovesicles).

The nanovesicles were stabilized by cross‐linking the membrane with EDC at 4 °C for 12 h for stabilization. Then, they were purified by ultrafiltration to remove excess EDC and free polymer. The cross‐linked nanovesicles formed by PEG‐PLys_75_‐PAsp_130_ at 10 mg mL^−1^ showed high stability against physiological salt conditions, as determined by DLS (**Figure** **3**a). Moreover, the zeta‐potential was −7.1 mV in 10 mm phosphate buffer (pH 7.4) (Figure 3b). Cryo‐TEM observation of the sample confirmed the formation of unilamellar nanovesicles. (**Figure** **4**a). The size by cryo‐TEM was determined to be 127.4 ± 26.1 nm. Moreover, the membrane thickness was 14.7 ± 2.8 nm (Figure 4b,c), which corresponds to an unilamellar structure.

**Figure 5:**
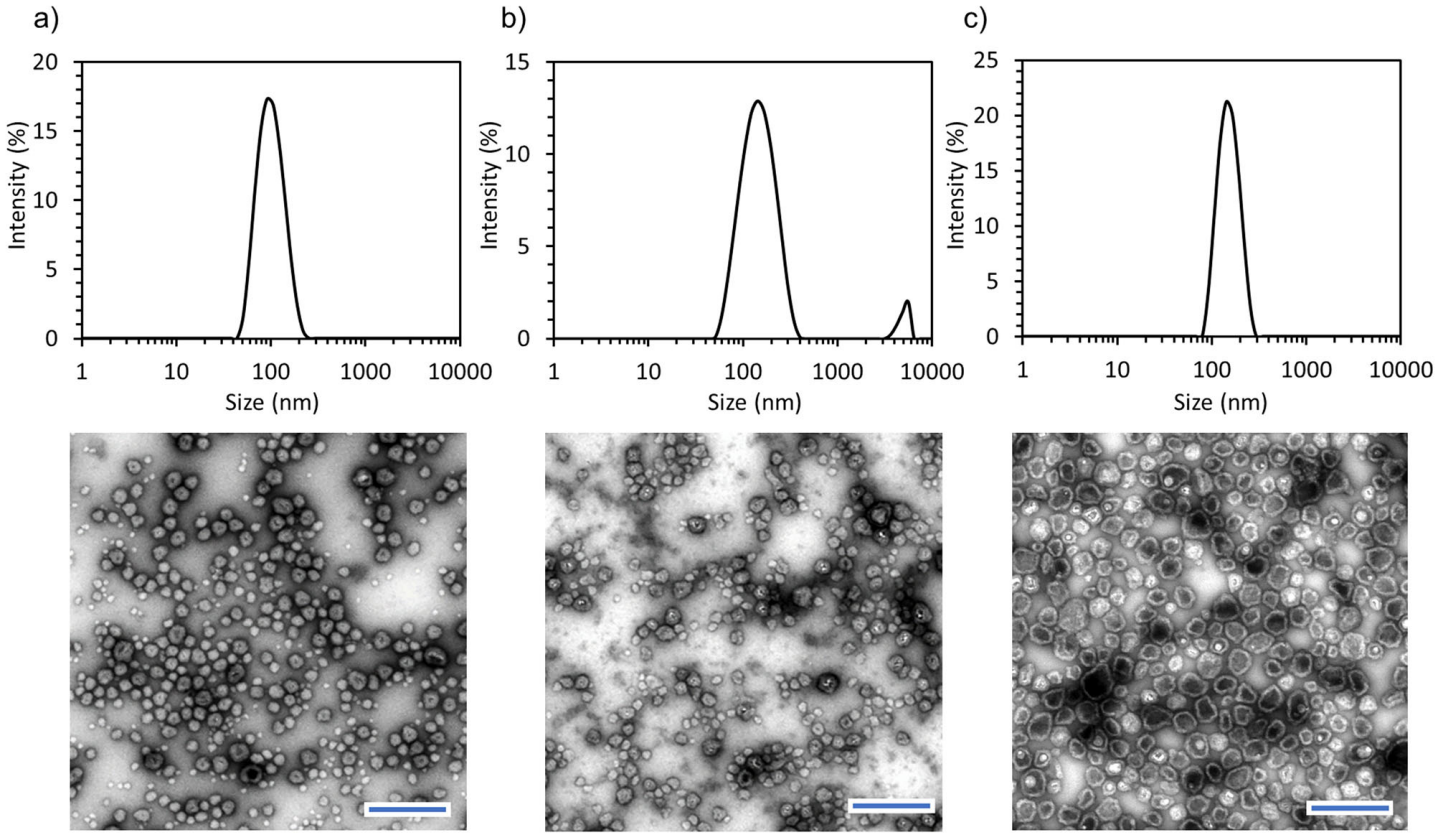
Representative TEM images and DLS histograms of particles prepared at polymer concentrations of a) 1 mg mL^−1^, b) 5 mg mL^−1^, and c) 10 mg mL^−1^, after cross‐linking by EDC at a C/A ratio of 1.7. All scale bars are 500 nm.

The cross‐linking process was used to study the effect of the polyampholyte concentration on the assembly of nanovesicles. Solutions of PEG‐PLys_75_‐PAsp_130_ in 10 mm phosphate buffer (pH 7.4) at concentrations of 1, 5, and 10 mg mL^−1^ led to the formation of nanostructures (**Figure** **5**a–c). The particle size increased from 100 to 145 nm, while the PDI decreased from 0.13 to 0.06 as the polymer concentration increased (Figure 5a,c). Although small particles of ≈30–40 nm were observed in the TEM images at lower concentrations (Figure 5a), monodisperse nanovesicles were observed at 10 mg mL^−1^ (Figure 5c). This heterogeneity at lower concentrations may result from a subset of the particle population, such as core–shell micelles.^[^ ^30^ ^]^ However, TEM analysis revealed that the vesicular structure was only achieved at a polymer concentration of 10 mg mL^−1^, and a mixture of small complexes and vesicles was observed at 1 and 5 mg mL^−1^ concentrations. The observed structure from TEM is consistent with previously reported polymersome structures.^[^ ^31^, ^32^ ^]^

The stability of the cross‐linked vesicles was further evaluated by incubation in D‐PBS containing 10% FBS at 37 °C for 24 h. The results showed that the size and the PDI remained comparable throughout the experiment for more than 24 h (Figure S7, Supporting Information). These findings suggest the suitable stability of the vesicles for challenging in vivo settings due to the presence of a dense PEG shell, which effectively mitigates serum adsorption^[^ ^33^ ^]^ and could reduce the formation of a protein corona.^[^ ^34^ ^]^

### 3.4 Evaluation of Blood Circulation and Biodistribution

**Figure 6:**
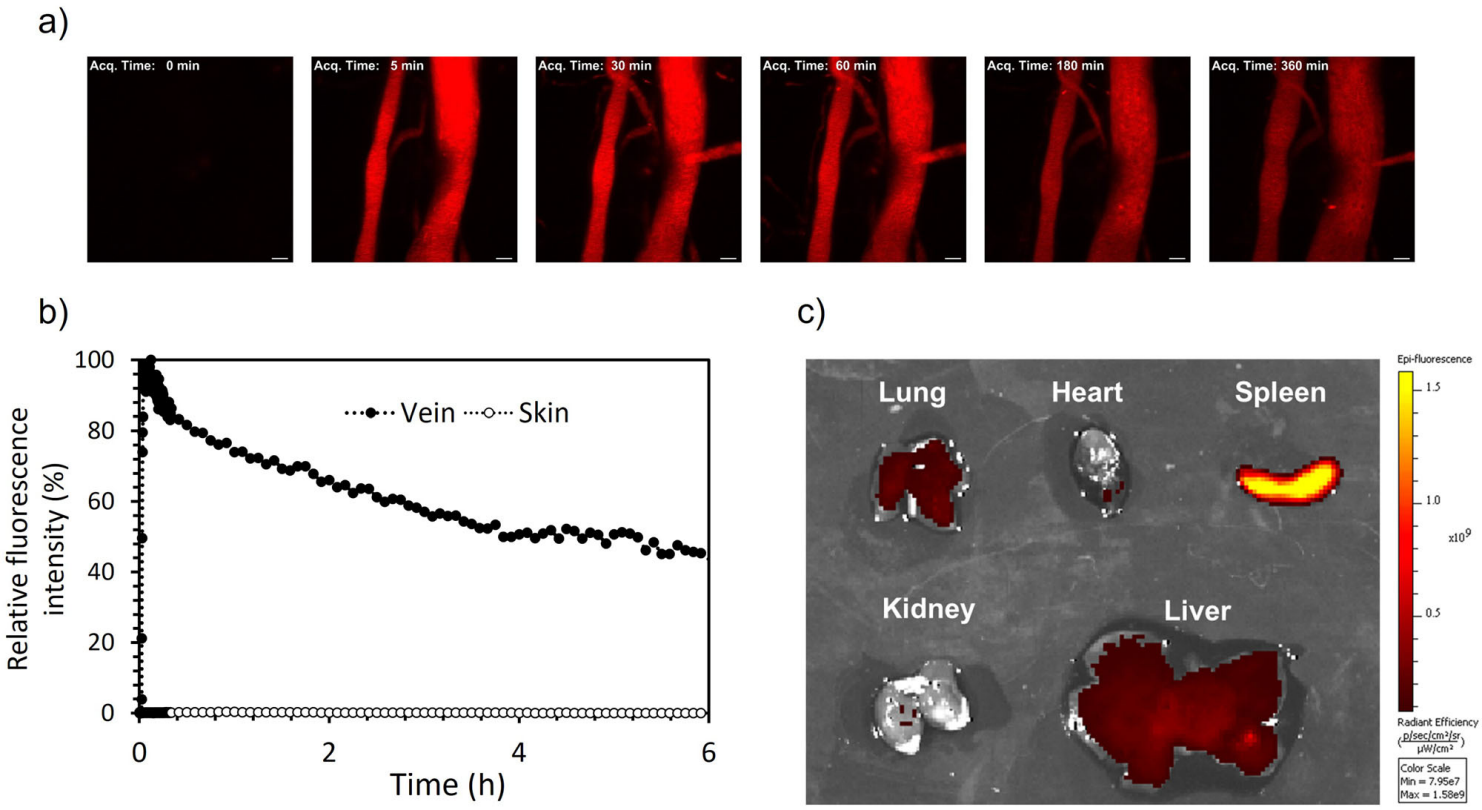
Blood circulation profile and biodistribution of Cy5‐labeled nanovesicles. a) Captured microscopic images from IV‐CLSM after injection of the nanovesicles (Scale bar = 100 µm). b) The time profile of the fluorescence intensity in the blood vessels and the skin, normalized to the maximum fluorescence intensity in the blood vessel. c) Ex vivo fluorescence imaging of Cy5‐labeled nanovesicles in lung, heart, spleen, kidney, and liver was determined by IVIS.

To determine the ability of our platform as a drug delivery system, we evaluated the circulation of the cross‐linked nanovesicles in the bloodstream, as well as their distribution to organs in mice. For tracing the nanovesicles in vivo, we labeled them with Cy5 (Cy5‐TPBV) by conjugating fluorescent molecules to the P(Lys) of PEG‐PLys_75_‐PAsp_130._ GPC results showed the Cy5 labeling to TPBV (Figure S8, Supporting Information). The Cy5‐TPBV was intravenously (i.v.) injected into mice, and the fluorescent intensity in the blood vessels in the skin was continuously monitored by IV‐CLSM (**Figure** **6**a). The result shows that the nanovesicles have long blood circulation with a half‐life of around 4 h (Figure 6b). In the skin, the fluorescence signal of the nanovesicles was not detectable, indicating that their limited extravasation.

An ex vivo fluorescence imaging analysis of the organs was performed 12 h post‐injection to determine the biodistribution of the nanovesicles (Figure 6c). The results revealed selective accumulation of TPBV in the spleen, which corresponds with the expected behavior for PEGylated nanoparticles with a size of 150 nm.^[^ ^35^ ^]^ It is estimated that the selective accumulation of TPBV in the spleen, rather than the liver, is owing to its negatively charged surface, high PEG density resulting in low protein adsorption, and its particle size. In PIC‐based nanocarriers, negatively charged carriers have been reported to be less readily taken up by liver sinusoidal endothelial cells, likely due to electrostatic repulsion from the negatively charged endothelial surface.^[^ ^30^ ^]^ In addition, TPBV exhibits low protein adsorption, which we attribute to its high PEG density, as shown (Figure S7, Supporting Information). This characteristic likely prevents the adsorption of liver‐targeting proteins such as Apolipoprotein E, thereby reducing hepatic uptake.^[^ ^33^, ^36^, ^37^, ^38^ ^]^ Furthermore, the spleen contains discontinuous capillaries, and it has been shown that PIC‐type nanoparticles in the size range of 100–300 nm tend to accumulate more in the spleen.^[^ ^39^ ^]^ Based on these properties, we estimate that TPBV preferentially accumulates in the spleen over the liver. These findings indicate that the system could be promising for targeted drug delivery to the spleen.

Enzymes and quantum dots have been successfully encapsulated in nano‐PICsomes, and the system has been reported can allow target‐specific drug delivery.^[^ ^3^, ^40^, ^41^ ^]^ As the TPBV also presents a large internal space, it is expected to enable the selective delivery to the spleen and release of these drugs in target cells through the stabilization of the membrane by a cleavable linker.^[^ ^42^ ^]^ In future studies, we plan to evaluate the drug loading efficiency and the drug release profile of our TPBV. Given the cross‐linked nature of the nanocarriers, we hypothesize that the controlled release would depend on the degradation of the cross‐linking bonds, which could be designed to sense changes in pH or enzymatic activity. Understanding these release mechanisms will be crucial for optimizing the delivery of therapeutic agents. Thus, the next phase of our research will include detailed assessments of drug loading efficiency and release kinetics, providing valuable insights into the practical applications of our platform for drug delivery.

## 4 Conclusion

This study presents the synthesis and biological performance of TPBV. By modulating the C/A ratio of triblock polyampholytes composed of neutral PEG, cationic poly(L‐lysine), and anionic poly(aspartate) segments, we successfully engineered nanovesicles with a hydrodynamic diameter of around 140 and a 15 nm unilamellar membrane. In vivo experiments demonstrated prolonged blood circulation and selective accumulation in the spleen. These findings indicate TPBV as promising nanocarriers for applications requiring extended blood circulation and spleen‐specific accumulation.

## Conflict of Interest

The authors declare no conflict of interest.

## Supporting information



Supporting Information

## Data Availability

The data that support the findings of this study are available from the corresponding author upon reasonable request.
