# Supplementary material for: Triblock Polyampholyte‐Based Nanovesicles for Targeted Spleen Delivery
Source: Macromol Biosci. 2025 May 22;26(1):e00147. doi: 10.1002/mabi.202500147 (PMC12829516; doi:10.1002/mabi.202500147)
Supplement: Supplementary file 1 — Supporting Information [file MABI-26-e00147-s001.docx]

Supporting Information

**Triblock Polyampholyte-Based Nanovesicles for Targeted Spleen Delivery**

Takayoshi Watanabe, Keita Masuda, Pengwen Chen and Horacio Cabral*


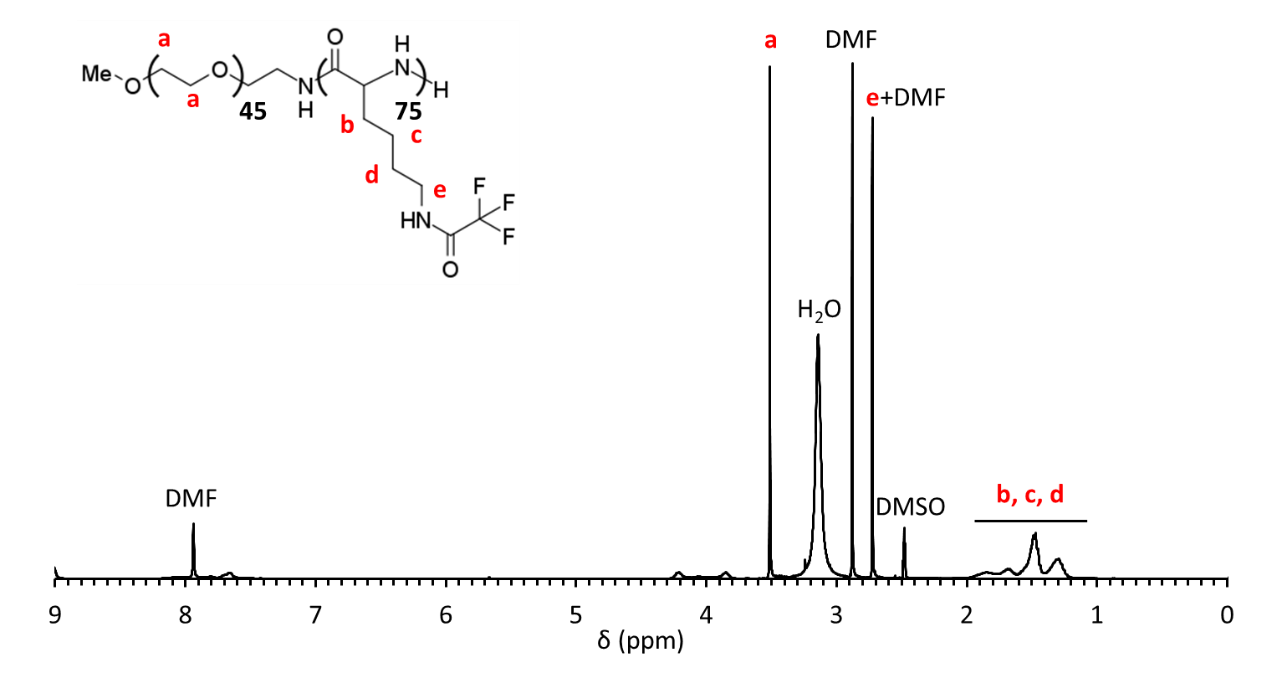


**Figure S1**. ^1^H-NMR spectrum of PEG-PLys(TFA)_75_. (solvent: DMSO-*d*_6_, temperature: 80 ℃.)


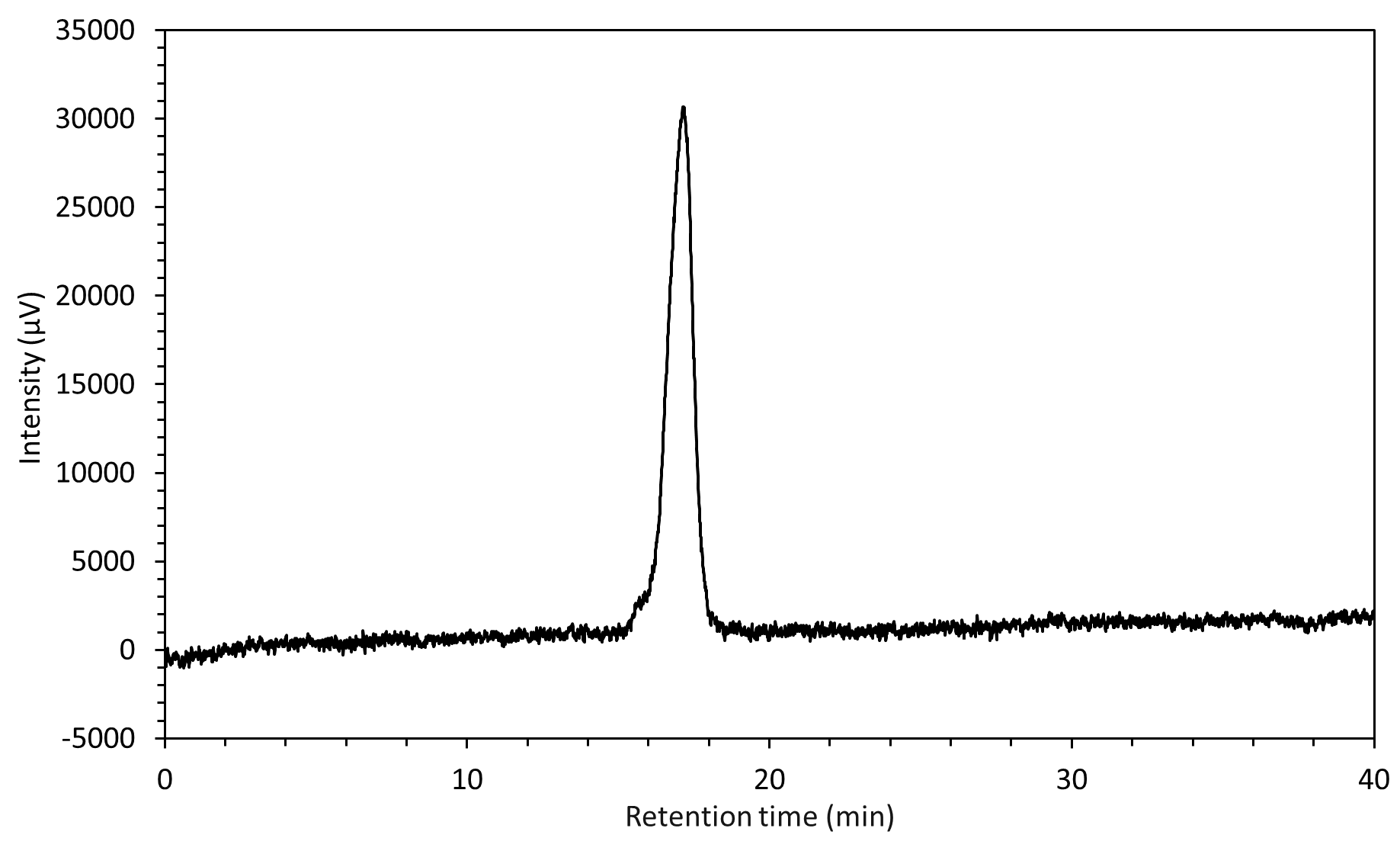


**Figure S2**. GPC profile of PEG-PLys(TFA) (1 mg mL^-1^, eluent: DMF with 10 mM lithium chloride, temperature: 40 °C, detector: UV at 220 nm, flow rate: 0.75 mL min^−1^).


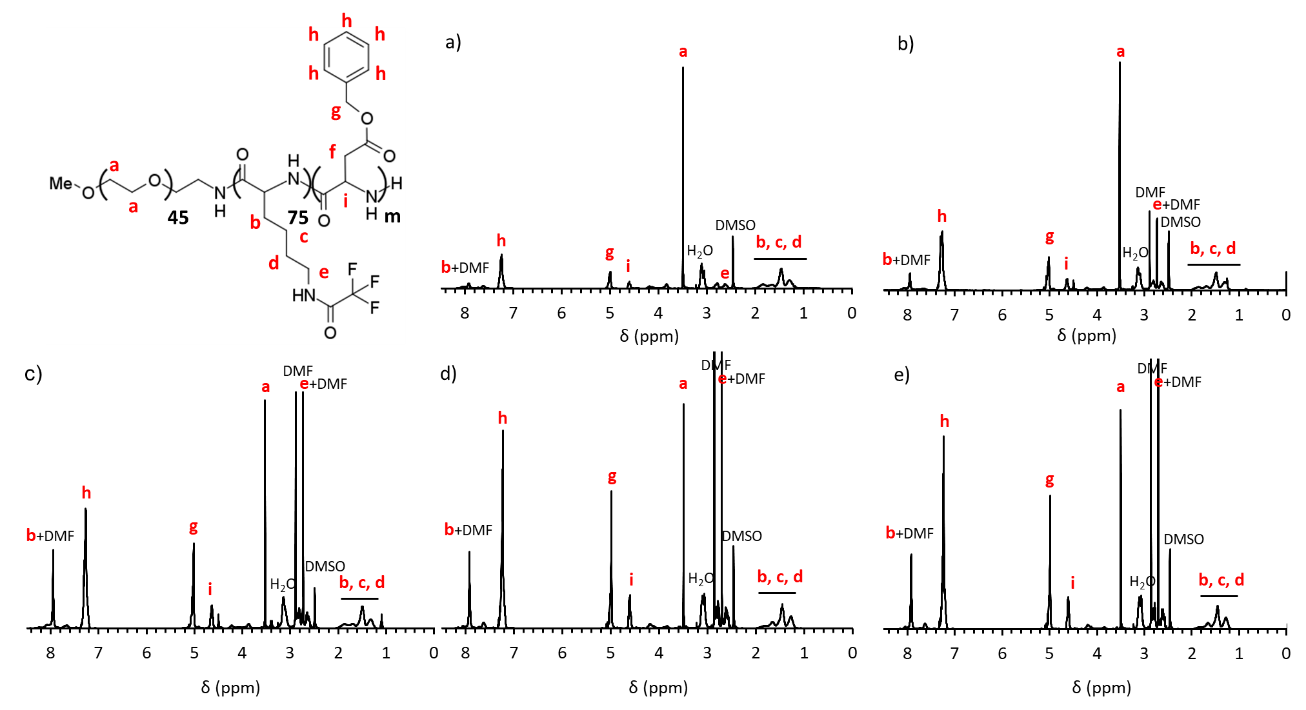


**Figure S3**. ^1^H-NMR spectra of a) PEG-PLys(TFA)_75_-PBLA_37_, b) PEG-PLys(TFA)_75_-PBLA_56_, c) PEG-PLys(TFA)_75_-PBLA_92_, d) PEG-PLys(TFA)_75_-PBLA_130_ and e) PEG-PLys(TFA)_75_-PBLA_170_. (solvent: DMSO-*d*_6_, temperature: 80 ℃.)


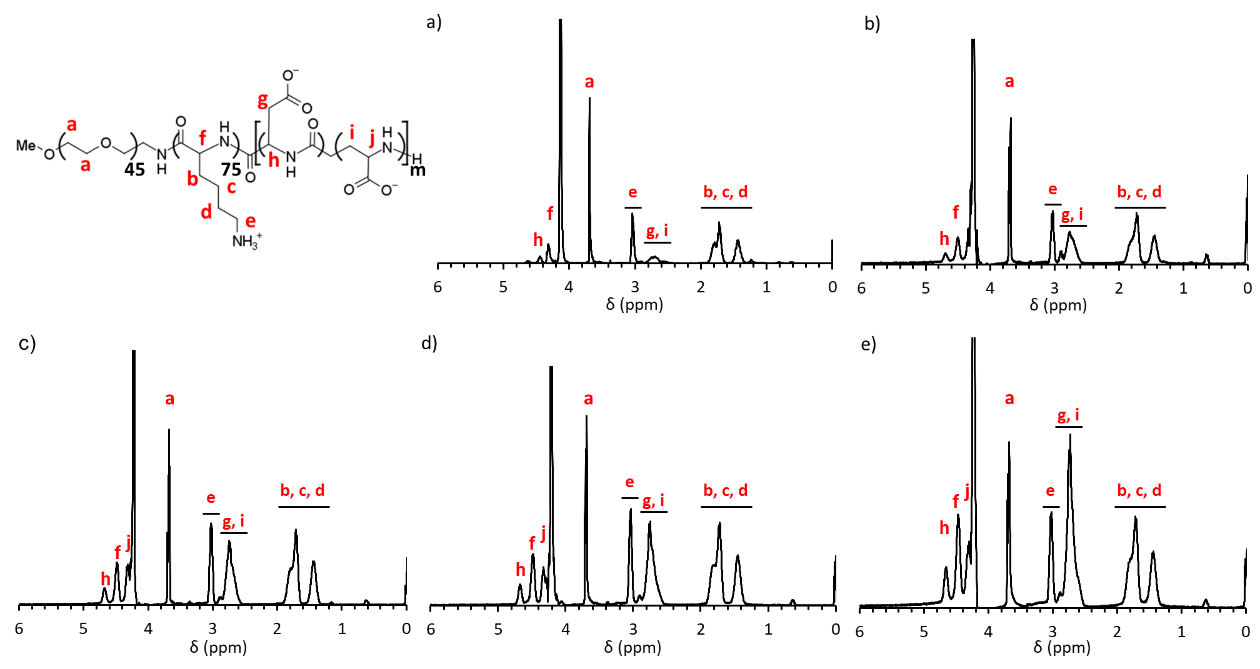


**Figure S4.** ^1^H-NMR spectra of a) PEG-PLys_75_-PAsp_37_, b) PEG-PLys_75_-PAsp_56_, c) PEG-PLys_75_-PAsp_92_, d) PEG-PLys_75_-PAsp_130_ and e) PEG-PLys_75_-PAsp_170_. (solvent: D_2_O plus 2 M NaCl, temperature: 80 ℃.)


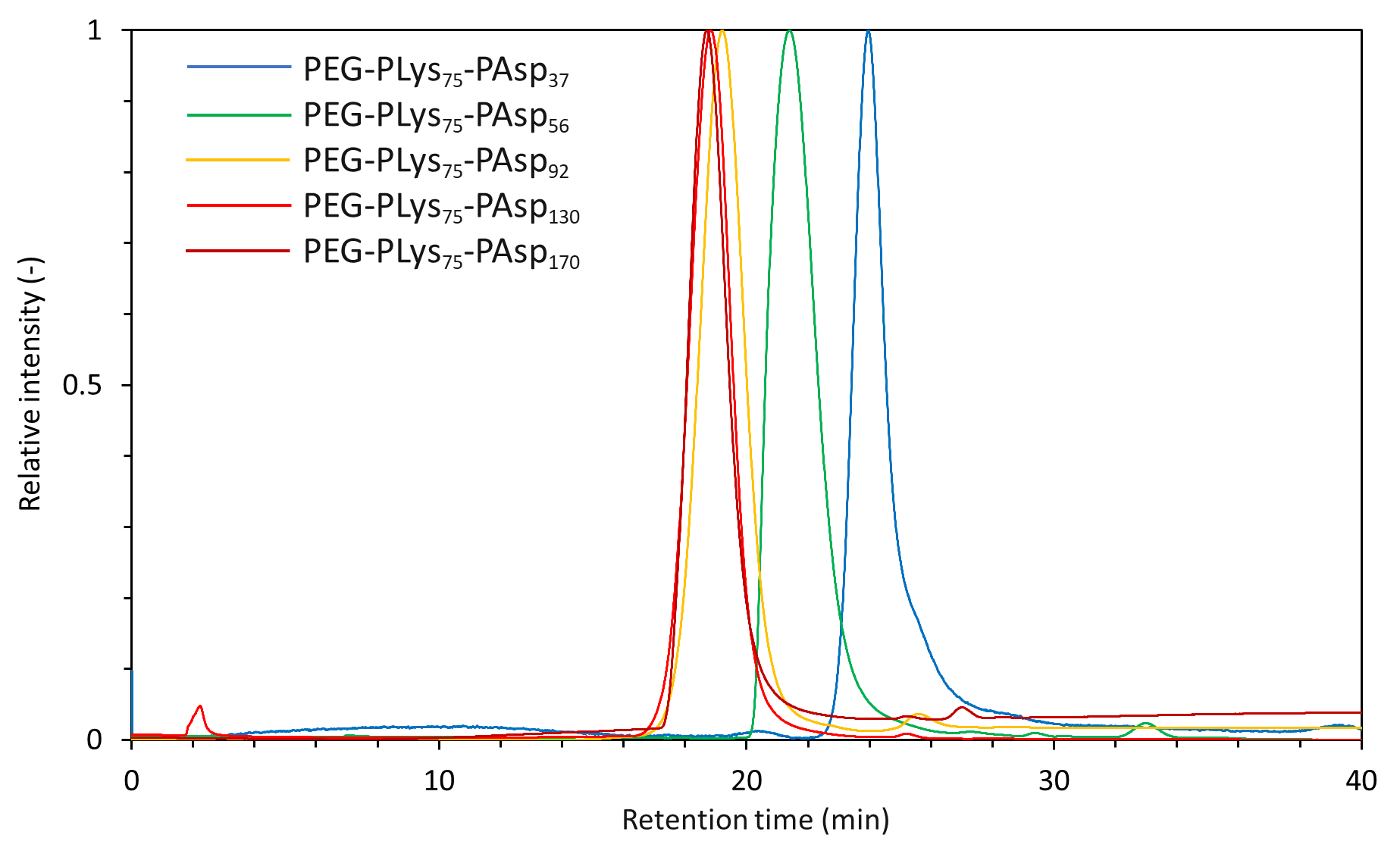


**Figure S5.** GPC profile of PEG-PLys-PAsp (eluent: 10 mM PBS buffer; pH 7.4, 2.0 M NaCl; room temperature, detector: UV at 220 nm, flow rate: 0.75 mL min^−1^).


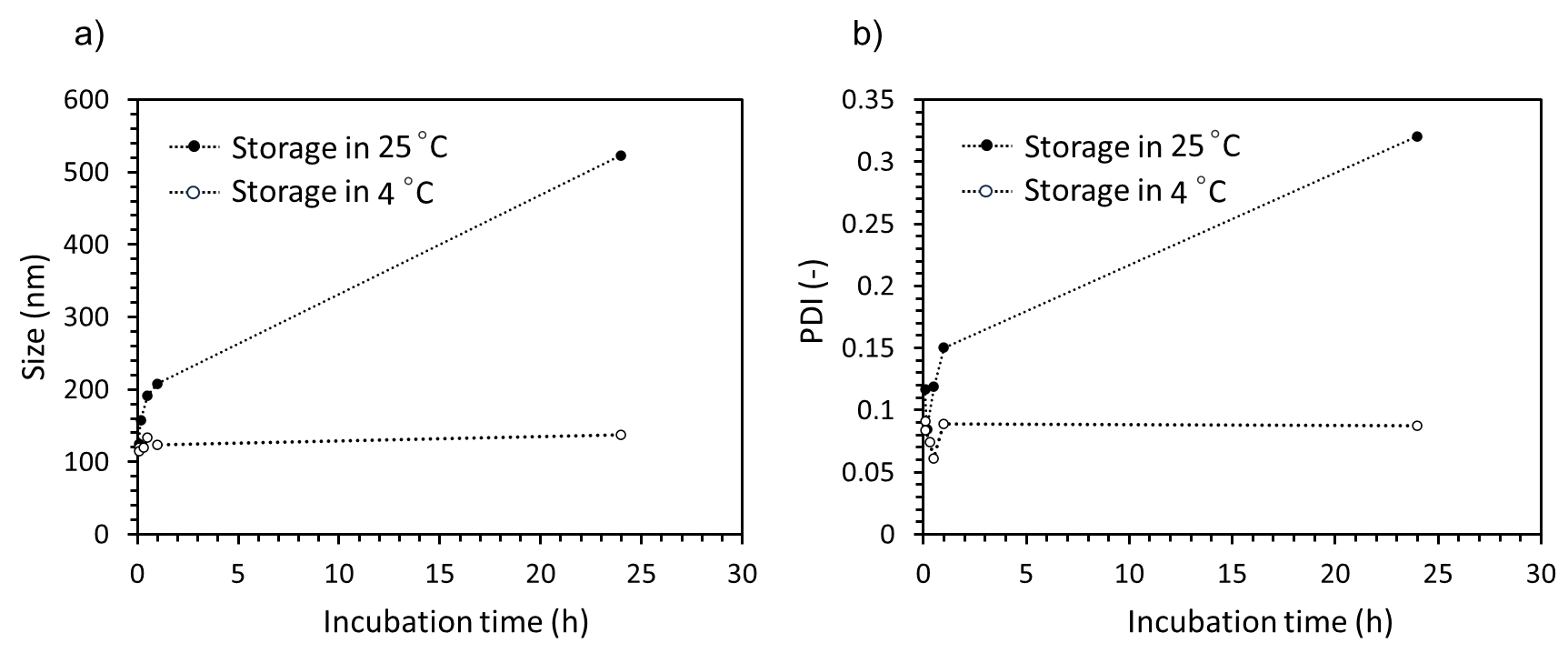


**Figure S6.** Stability of vesicle before crosslinking in 10 mM PB buffer (pH7.4) at different temperatures from a) size and b) polydispersity index (PDI).


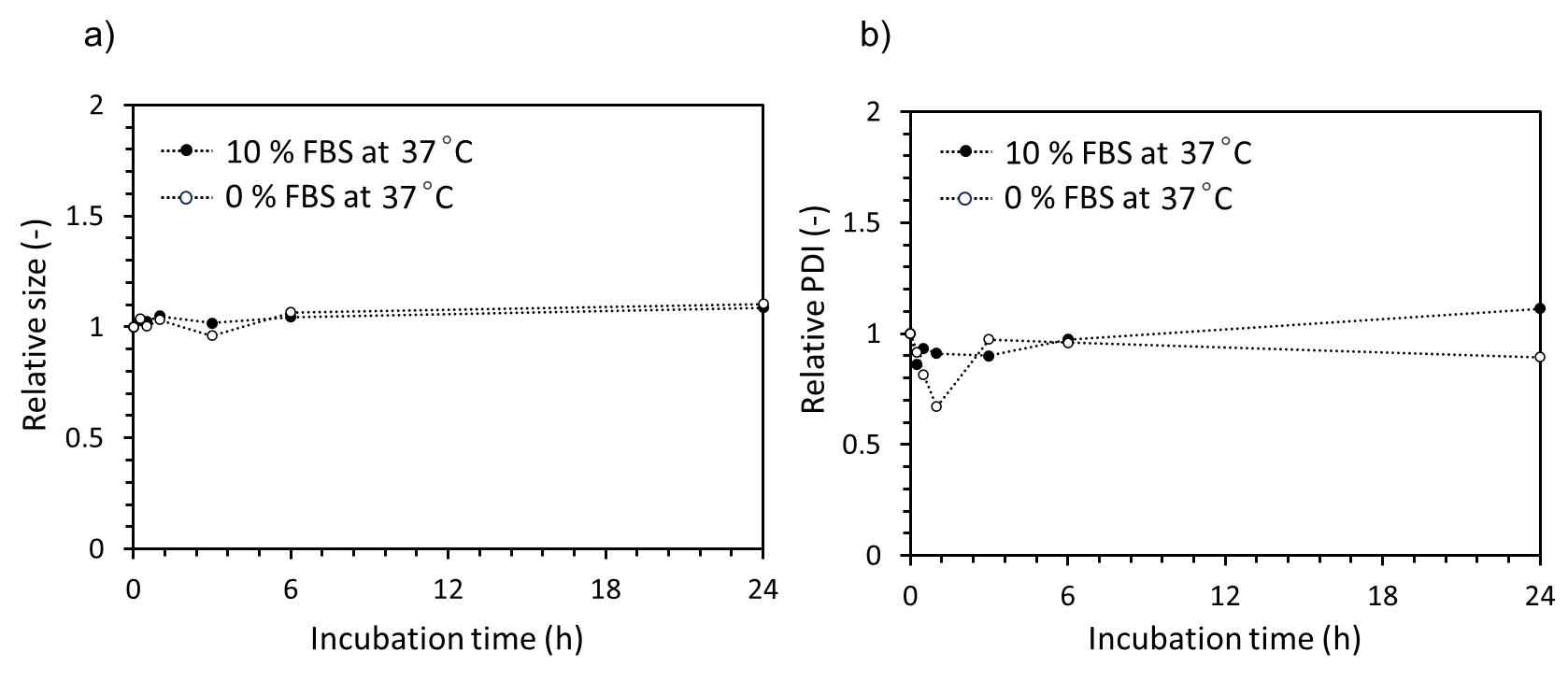


**Figure S7.** FBS stability of vesicle after crosslinking in D-PBS at different FBS concentrations, evaluated by (a) relative size and (b) PDI compared to the initial incubation time.


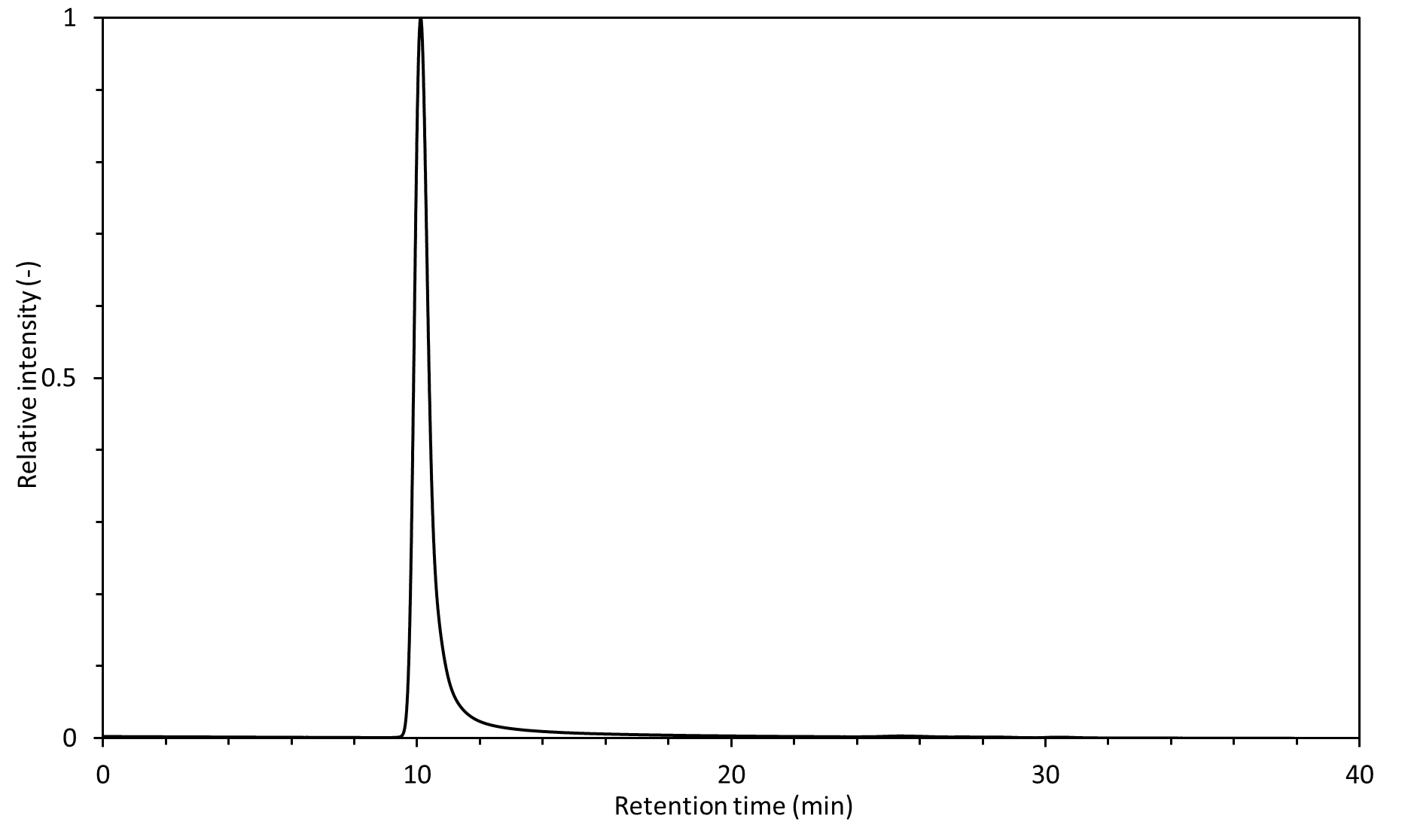


**Figure S8.** GPC profile of Cy5-TPBV (eluent: 10 mM PBS buffer; pH 7.4, 500 mM NaCl; room temperature, detector: E_x_640/E_m_680, flow rate: 0.75 mL min^−1^). The result indicates the absence of free polymers.
